# *BRAF* mutations, microsatellite instability status and cyclin D1 expression predict metastatic colorectal patients’ outcome

**DOI:** 10.1038/sj.bjc.6605694

**Published:** 2010-05-18

**Authors:** Z Saridaki, D Papadatos-Pastos, M Tzardi, D Mavroudis, E Bairaktari, H Arvanity, E Stathopoulos, V Georgoulias, J Souglakos

**Affiliations:** 1 Laboratory of Tumor Cell Biology, School of Medicine, University of Crete, Crete, Greece; 2 Department of Medical Oncology, University General Hospital, Heraklion, Crete, Greece; 3 Laboratory of Pathology, University General Hospital, Heraklion, Crete, Greece

**Keywords:** *BRAF* mutations, MSI status, cyclin D1 expression, metastatic CRC

## Abstract

**Background:**

The significance of *BRAF* mutations, microsatelite instability (MSI) status and cyclin D1 expression in patients with metastatic colorectal cancer (mCRC) was evaluated.

**Methods:**

Primary tumours from 144 patients treated for mCRC were assessed for *BRAF* (V600E) mutation, MSI status and cyclin D1. The data were correlated with progression-free survival (PFS) and overall survival (OS).

**Results:**

*BRAF* mutations were detected in 10 (out of 22, 45%) patients with MSI-H tumours compared with 2 (out of 122, 1.6%) in those with microsatellite stable tumours (*P*<0.001). The presence of *BRAF* mutations was correlated with cyclin D1 overexpression (7 out of 26 patients, 58% *vs* 5 out of 118 patients, 14% *P*=0.001). Patients with *BRAF*-mutated primary tumours had a significantly decreased PFS (2.7 *vs* 9.8 months; *P*<0.001) and median OS (14 *vs* 30 months; *P*<0.001) than patients with wild-type (wt) tumours. Patients with MSI-H and *BRAF*-mutated tumours experienced significantly lower PFS (3.1 *vs* 11.4 months; *P*=0.008) and OS (14.5 *vs* 35.5 months; *P*=0.004) than patients with MSI-H and *BRAF* wt tumours. Similarly, *BRAF* mutations and cyclin D1 overexpression were correlated with decreased PFS (3.1 *vs* 8.6 months; *P*=0.03) and OS (17.8 *vs* 39.2 months; *P*=0.01).

**Conclusion:**

*BRA*F V600E mutations are associated with MSI-H status and cyclin D1 overexpression and characterize a subgroup of patients with poor prognosis.

Colorectal cancer (CRC) remains a major public health problem in the Western world with an estimated 146 970 new cases and 49 920 deaths in the United States in 2009 (Jemal *et al*, 2009). Some molecular markers have already been incorporated in the treatment of CRC patients. Indeed, the knowledge of *KRAS* mutational status of a primary tumour is now mandatory for the treatment of metastatic disease, as it is a predictor of resistance to monoclonal antibodies of the epidermal growth factor receptor (anti-EGFR moAbs) (Bokemeyer *et al*, 2008; Douillard *et al*, 2009; Hecht *et al*, 2009; Tol *et al*, 2009; Van Cutsem *et al*, 2009). In addition, *BRAF* V600E mutation identifies a subgroup (<10%) of patients with unfavourable prognosis (Di Nicolantonio *et al*, 2008; Souglakos *et al*, 2009); conversely, the presence of a defective tumoural DNA mismatch repair (dMMR) system seems to be a favourable prognostic factor (French *et al*, 2008), although these patients seem to respond worse to standard adjuvant chemotherapy (Hemminki *et al*, 2000; Samowitz *et al*, 2001; Popat *et al*, 2005; French *et al*, 2008).

The RAS–RAF–MEK–ERK–MAP kinase pathway mediates cellular responses to growth signals constituting an essential component of intracellular signalling from activated cell-surface receptors to transcription factors in the nucleus (Peyssonnaux and Eychene, 2001). In the *BRAF* gene, one of the three *RAF* genes and part of the above-mentioned pathway, the single substitution missense mutation V600E, located within the kinase domain, account for more than 80% of the described mutations. The *BRAF* V600E mutation has been detected in a wide range of human cancers, including melanomas, thyroid carcinomas, sporadic CRC (10%) and others. The V600E mutation results in a constitutive activation of the BRAF kinase promoting cell transformation (Davies *et al*, 2002; Preto *et al*, 2008).

In a retrospective study of sporadic metastatic colorectal cancer (mCRC), *BRAF* mutations, detected in 8% of patients, were emerged as an independent prognostic factor for both progression-free survival (PFS) and overall survival (OS) (Souglakos *et al*, 2009). Similar findings were reported by Di Nicolantonio *et al* (2008) who concluded that the *BRAF* V600E mutation is not only inversely associated with response to anti-EGFR moAb therapy, but it is also associated with a worse prognosis.

The microsatellite instability (MSI), which is characterised by the absence of protein expression encoded by the corresponding MMR genes (*hMLH1*, *hMSH2*, *hMSH6* or *PMS2;* Thibodeau *et al*, 1993, 1998; Popat *et al*, 2005), is observed in nearly all patients with CRC due to hereditary non-polyposis colon cancer (HNPCC) (Aaltonen *et al*, 1993, 1994; Thibodeau *et al*, 1993) and in 15–20% of patients with sporadic CRC (Aaltonen *et al*, 1993). In its familial form, the genetic basis of instability is mainly (80%) inherited germ-line mutations of the *MMR* genes (especially *hMLH1* and *hMSH2*; Leach *et al*, 1993; Peltomaki, 1994), whereas in the sporadic form, it is due to *hMLH1* inactivation by epigenetic hypermethylation of the promoter and less frequently to genetic alterations of *hMSH2* and *hMSH6* genes (Kane *et al*, 1997; Cunningham *et al*, 1998, 2001; Thibodeau *et al*, 1998). Microsatellite genotyping of CRC patients for clinically applicable diagnosis is based on specific standard criteria using specific panels (Boland *et al*, 1998). Since then, these panels have been clinically applicable for the diagnosis of CRC patients (Gryfe *et al*, 2000; Watanabe *et al*, 2001).

In CRC tumours, *BRAF* mutations seem to occur more frequently in cases characterised by dMMR (Rajagopalan *et al*, 2002). Moreover, several studies suggest that the *BRAF* V600E mutation occurs much more frequently in MSI-H tumours in comparison with microsatellite stable (MSS) tumours (50 *vs* <5% respectively) (Wang *et al*, 2003; Domingo *et al*, 2004).

Cyclin D1 is a cell-cycle regulatory protein and its upregulation has been associated with increased proliferation and poor clinical outcome in various tumours (Le Marchand *et al*, 2003). Cyclin D1 is a key element in the downstream EGFR signalling pathway; *KRAS* mutations lead to the activation of the RAS–RAF–MEK–ERK–MAP kinase pathway by inducing cyclin D1 synthesis (Kobayashi *et al*, 2006). *BRAF* controls proliferation of human melanoma cells through the regulation of cyclin D1 and cyclin-dependent kinase inhibitor p27^Kip1^ protein (Bhatt *et al*, 2005, 2007). Similarly, it has been reported that in CRC cells the decreased expression levels of pERK protein and cyclin D1 were more pronounced in cells carrying the *BRAF* V600E mutation, and, that *BRAF* V600E–ERK signalling is also important in the regulation of proliferation by p27^Kip1^ and cyclin D1 proteins in these cells (Preto *et al*, 2008).

The aim of this study was to investigate the clinical relevance of *BRAF* V600E mutation status, cyclin D1 expression and MSI status of primary tumours of patients with mCRC treated with front-line 5FU-based chemotherapy.

## Materials and methods

### Patient population and study design

A total of 144 consecutive patients, with histologically confirmed mCRC and available tumour material for molecular analysis, who were treated at the University Hospital of Heraklion (Crete, Greece) between January 2002 and December 2006 were enrolled. The study was approved by the institutional ethics committee and all patients gave the informed consent for the use of the tissue material for translational research.

The majority of patients were treated in the context of two clinical trials conducted at our centre (Souglakos *et al*, 2006; Emmanouilides *et al*, 2007; Tables 1 and 2). Patients were evaluated at baseline and every four cycles of chemotherapy. Disease status was coded, without the knowledge of the laboratory analysis.

### Tissue selection and DNA extraction

Formalin-fixed, paraffin-embedded tumour sections were reviewed by a pathologist (MT) to confirm the diagnosis and define tumour-enriched areas for dissection. Ten serial sections of 5 *μ*m thickness were stained with nuclear fast red (Sigma-Aldrich, St Louis, MO, USA) and scrape dissection under a binocular microscope was performed for samples with ⩾80% tumour cells; for samples with <80% malignant cells, microdissection with the piezoelectric Eppendorf microdissector (Eppendorf, Hamburg, Germany) was performed. Isolated cancer cells were lysed in buffer containing Proteinase K at 60°C for 72 h, followed by DNA extraction using the MasterPure Complete DNA and RNA Purification Kit according to the manufacturer's instructions (Epicentre Biotechnologies, Madison, WI, USA).

### *BRAF* testing

The V600E *BRAF* mutation was detected by real-time PCR using the allelic discrimination method as previously described (Benlloch *et al*, 2006). In brief, the DNA extracted from tumoural cells was amplified with the use of a set of primers and two hydrolysis probes in the ABI PRISM 7900T Sequence Detection System (Applied Biosystems, Forest City, CA, USA). The two hydrolysis probes were labelled at 5 with VIC and FAM fluorophores reporters for the wild-type (wt) and the mutant allele respectively. The SDS 2.3 software (Applied Biosystems) was used for the analysis of the results.

### Immunohistochemistry for MMR and cyclin D1

Tumour sections form each patient were selected for immunohistochemical staining using anti-hMLH1 and anti-hMSH2 (for the MMR definition as previously described; Lindor *et al*, 2002) and anti-cyclin D1 (Nosho *et al*, 2008) antibodies. In brief, immunostaining was performed using the UltraVision LP Large Volume Detection System AP Polymer (Thermo Scientific, Waltham, MA, USA). The primary antibodies and their corresponding dilutions used were: hMLH1 (Cell Marque, Rocklin, CA, USA; dilution 1 : 50), hMSH2 (Cell Marque Rocklin; dilution 1 : 50) and Cyclin D1 (Neomarker, Fermont, CA, USA; dilution 1 : 25). Nuclear immunostaining of lymphocytes and stromal cells served as internal positive control for hMLH1 and hMSH2. Tumours showing loss of nuclear hMSH2 or hMLH1expression were classified as hMLH1 or hMSH2 negative. In addition, for cyclin D1 a positive control slide from a case of mantle cell lymphoma was included and nuclear immunostaining was considered positive. Negative control slides were prepared by omitting the primary antibody. Nuclear cyclin D1 expression was recorded as no expression, weak expression or moderate/strong expression. Cyclin D1 overexpression was defined as ⩾50% of tumour cells with weak nuclear staining or ⩾20% of tumour cells with moderate/strong nuclear staining (Nosho *et al*, 2008).

### MSI testing and mismatch repair definition

Microsatelite instability status was evaluated in all samples using the five microsatellite markers of the NCI reference panel (BAT-25 at 4q12, BAT-26 at 2p16, D2S123 at 2p16-p21, D5S346 at 5q21-q22 and D17S250 at 17q11.2-q12) and with two additional microsatellite markers of the alternative panel (D18S58 at 18q22-q23 and D18S61 at 18q22) (Boland *et al*, 1998). PCR for the above-mentioned microsatellite markers was carried out on tumour and matched DNA from the adjacent normal colonic tissue. Standard PCR conditions were used and included 10 × buffers, Taq gold and deoxynucleotide triphosphates adjusted to a final reaction volume of 25 *μ*l containing 100 ng of genomic DNA (Boland *et al*, 1998; Watanabe *et al*, 2001).

Single-strand conformation polymorphism in non-denaturing environment (Makino *et al*, 1992) was performed after optimisation with a 7% polyacrylamide gel (5% glycerol) for the analysis of BAT-25 and BAT-26 PCR products. The PCR products of other microsatellite markers’ were analysed in 7% polyacrylamide gels and silver-stained. Gels were scanned and the intensity of the bands corresponding to the microsatellite alleles was quantified by a digital image analysis system, as previously described (Saridaki *et al*, 2003). The analysis was repeated at least twice and the same results were obtained in all cases. MSI was diagnosed in case of an addition or deletion of one or more repeat units resulting in novel alleles. All the heterozygous cases, and those that were constitutionally homozygous (non-informative) for a marker, were counted to estimate the MSI rate. If ⩾30% of the loci examined showed MSI, the tumour was classified as MSI-H. If <30% of the loci examined showed MSI, it was classified as MSI-L and if none of the examined loci showed instability, the tumour was classified as MSS (Boland *et al*, 1998).

### Statistical analysis

Associations between *BRAF* mutation status, dMMR, cyclin D1 expression and baseline characteristics were assessed using the Fisher's exact test for categorical variables or logistic regression for continuous variables. PFS was measured from the date of first-line therapy initiation to the first radiographic documentation of disease progression or death, and OS was calculated from the date of diagnosis of metastatic disease to death due to mCRC. Kaplan–Meier curves were used to describe the proportion of patients who remained free of events over the follow-up period. Associations between prognostic factors and PFS or OS were examined using Cox proportional hazards regression models. All reported *P*-values are two sided and not adjusted for multiple testing.

## Results

**Figure 1:**
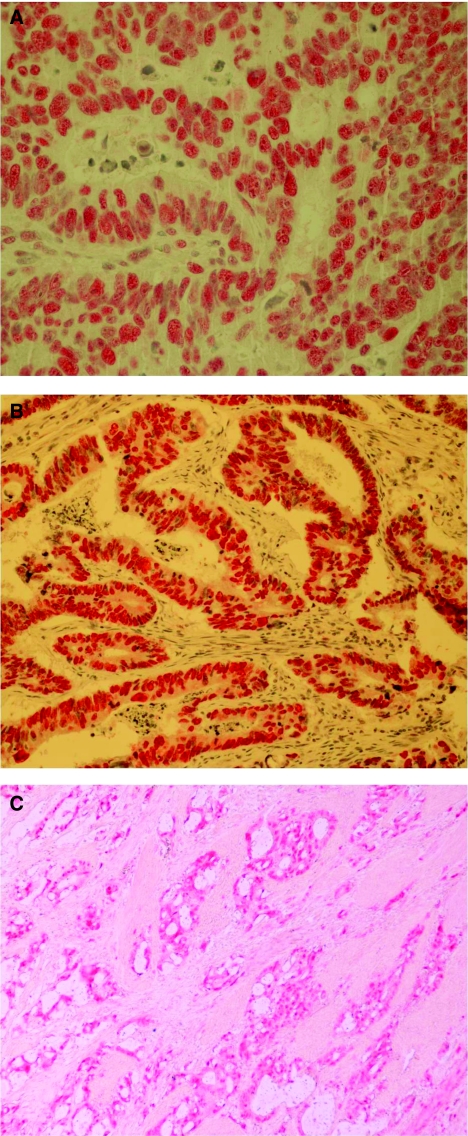
(**A**) MLH1(+) adenocarcinoma with moderate differentiation of the colon (original magnification, × 200). (**B**) MSH2(+) adenocarcinoma with moderate differentiation of the colon (original magnification, × 100). (**C**) Cyclin D1( +) adenocarcinoma moderate–poorly differentiated (original magnification, × 100).

The median age of patients was 64 years and 57% of them were men. Metastasectomy was also performed in 21 (15%) patients (Tables 1 and 2). The *BRAF* V600E mutation was detected in 12 (8%) patients and 22 (15%) tumours were characterised as MSI-H. MSI analysis using immunohistochemistry and molecular techniques presented 100% accordance (Figure 1A and B). Cyclin D1 was overexpressed in 26 (18%) patients, weakly expressed in 63 (44%) and not expressed in 55 (38%) (Figure 1C). There was no correlation between the presence of *BRAF* mutation, MSI-H and cyclin D1 expression, and the patient’ gender, age, stage at diagnosis, histological grade and tumour location (all *P*-values >0.05).

The median time from initial diagnosis to diagnosis of metastatic disease was 19.3 months (95% CI 14.6–20.3) for patients with early stage disease and the median interval from the diagnosis of metastatic disease to treatment initiation 0.8 months (95% CI 0.5–1.1). All patients were treated with 5-FU-based first-line chemotherapy with or without moAb supplementation (Table 3). At the time of analysis 132 out of 144 (92%) patients were dead, 128 (97%) of them due to disease progression, 2 (1.5%) due to toxicity and 2 (1.5%) due to reasons unrelated with disease or treatment.

*BRAF* mutations were present in 45 and 1.6% of the patients with MSI-H and MSS tumours respectively (*P*<0.001). The detection of *BRAF* mutations was also correlated with cyclin D1 expression as cyclin D1 overexpression was detected in 58 and 14% of *BRAF* mutated and wt tumours respectively (*P*=0.001; Table 4).

**Figure 2:**
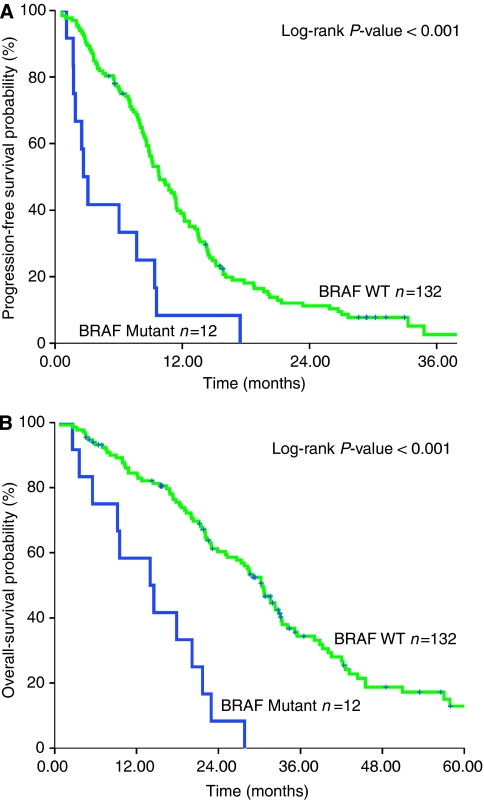
(**A**) Progression-free survival in first-line chemotherapy, analysed by *BRAF* mutation status. (**B**) Overall survival, analysed by *BRAF* mutation status.

The median PFS of the whole group of patients was 9.5 months (95% CI 8.4–10.8) and the corresponding median OS was 31.5 months (95% CI 26.4–37.7). The median OS was 14 and 30 months for patients with *BRAF*-mutated and wt tumours respectively (*P*<0.001; Figure 2A). In addition, PFS was 2.7 and 9.8 months for patients with *BRAF*-mutated and wt primary tumours respectively (*P*<0.001; Figure 2B). Patients with MSI-H and *BRAF*-mutated tumours experienced significantly lower PFS (3.1 *vs* 11.4 months; *P*=0.008) and OS (14.5 *vs* 35.5 months; *P*=0.004) in comparison with those with MSI-H and *BRAF* wt tumours. Similarly, *BRAF* mutations and cyclin D1 overexpression were correlated with decreased PFS (3.1 *vs* 8.6 months; *P*=0.03) and OS (17.8 *vs* 39.2 months; *P*=0.01).

Univariate analysis (Tables 1 and 2) revealed significant associations of PFS with undifferentiated tumour histology (*P*=0.001), *BRAF* mutations (*P*<0.001) and inability of patients to undergo metastasectomy (*P*<0.001). In addition, univariate analysis showed significant associations between OS and (1) tumour differentiation (grade 3) (PFS and OS: *P*<0.001); (2) *BRAF* mutations (*P*<0.0001), (3) metastasectomy (OS: *P*=0.03) and (4) the sum of treatment lines that a patient had the opportunity to receive (*P*=0.02).

In multivariate analysis, *BRAF* mutation and tumour grade were emerged as independent prognostic factors for reduced PFS (HR 2.8, 95% CI 1.4–5.7, *P*=0.004 and HR 2.0, 95% CI 1.3–3.2, *P*=0.001 respectively) and OS (HR 5.3, 95% CI 2.5–11.3, *P*<0.001 and HR 2.6, 95% CI 1.6–4.4, *P*<0.001 respectively). In addition, both metastasectomy and the number of administered treatment lines emerged as independent factors associated with increased PFS and OS (Table 5).

In addition, 48 (33%) patients were treated with cetuximab, 11 in the 1st line setting and 37 in 2nd and further lines. *KRAS* mutational status was available in 42 of these patients and 13 (31%) of them carried a mutation in their primary tumours. *KRAS* and *BRAF* mutation were found to be mutually exclusive. *KRAS* status predicted resistance to cetuximab therapy in terms of decreased PFS (*P*=0.045) and mOS (*P*=0.007). Also, four (8.5%) patients were found to harbour a *BRAF*^V600E^ mutation in the primary tumours. Similarly, patients with a *BRAF* mutation presented lower PFS (*P*=0.05) and mOS (*P*=0.004). When analysed together, the presence of either mutation was significantly correlated with decreased PFS (*P*=0.013) and mOS (*P*=0.003) in cetuximab-treated patients. *BRAF* mutation retains its prognostic significance in PFS (0.003) and mOS (<0.001) in the subpopulation of patients that have not received cetuximab in the course of their disease.

## Discussion

The results of this study show that patients with *BRAF*-mutated tumours had a significantly lower median PFS and OS compared with patients with wt tumours. In addition, multivariate analysis revealed that the presence of the V600E *BRAF* mutation was established as an independent prognostic factor for reduced PFS and OS. Similar results have been previously reported regarding the prognostic of *BRAF* mutation in patients with mCRC (Samowitz *et al*, 2005; Souglakos *et al*, 2009). In this study, median OS was 31.5 months, which is higher than survival reported by other studies. This improved OS could be related to the fact that after initial response to systemic treatment, 24% of patients underwent metastasectomy; alternatively, we cannot exclude that this observation may be due the fact that 87% of the patients had received all three chemotherapeutic drugs in the course of their treatment (Grothey *et al*, 2004; Hurwitz *et al*, 2004) whereas 45% of patients had also received both moAbs as well.

In this study, the incidence of *BRAF* mutations was significantly higher in patients with MSI-H (45%) than with MSS tumours (1.6% *P*=<0.001), in agreement with the published evidence (Rajagopalan *et al*, 2002; Oliveira *et al*, 2003; Wang *et al*, 2003). Moreover, our patients with MSI-H and *BRAF*-mutated tumours experienced significantly lower PFS (*P*=0.008) and OS (*P*=0.004) in comparison with those with MSI-H and *BRAF* wt primary tumours. Similar results have been reported regarding the prognostic significance of *BRAF* mutations in patients with early stage CRC (Samowitz *et al*, 2005; French *et al*, 2008). In the French *et al* (2008) trial among patients with dMMR, *BRAF* wt cases had a significantly improved OS compared with those that were *BRAF* mutated (*P*=0.001). Samowitz *et al* (2005) showed that patients with dMMR tumours were associated with an excellent 5-year OS regardless of the *BRAF* V600E status. However, they have also reported that the *BRAF* mutation was associated with poor survival among the patients with MSS tumours (Samowitz *et al*, 2005). Nevertheless, the direct comparison of these studies is difficult because of differences in the studied population, and additional studies with larger groups of patients are required.

In this study, the detection of *BRAF* mutations was associated with cyclin D1 expression. Indeed, the incidence of cyclin D1 overexpression was significantly higher in *BRAF*-mutated (58%) than *BRAF* wt tumours (14% *P*=0.001), and patients with *BRAF* mutations and cyclin D1 overexpression had significantly decreased PFS (*P*=0.03) and OS (*P*=0.01) compared with patients with *BRAF* wt tumours. The biological significance of the *BRAF* V600E mutation and oncogenic activation in MSI-H CRC is not well established. The effect of BRAF knockdown in cellular survival and proliferation is not fully understood. It has been shown that in MSI CRC cell lines, *BRAF* is the main activator of ERKs and these cells are more dependant on the BRAF–ERK pathway (Preto *et al*, 2008). Similar to the melanoma model (Bhatt *et al*, 2005, 2007), in MSI CRC cells, it was shown that *BRAF* V600E–ERK signalling is important in the regulation of proliferation through the p27^Kip1^ and cyclin D1 proteins (Preto *et al*, 2008).

Furthermore, the presence of BRAF mutations has been correlated with resistance to the anti-EGFR moAb cetuximab (Di Nicolantonio *et al*, 2008; Souglakos *et al*, 2009). Moreover, the introduction/presence of the *BRAF* V600E allele in CRC (DiFi-BRAF, COLO-205 and HT-29) cell lines impaired the therapeutic potential of anti-EGFR moAbs. In contrast, when these cells lines were treated with a combination of cetuximab and the small-molecule kinase BRAF inhibitor, sorafenib, a significant reduction in proliferation and a prominent proapoptotic effect was found, whereas, either of these agents alone had limited effects (Di Nicolantonio *et al*, 2008). Thus, in the clinical setting, the therapeutic effect of anti-EGFR moAbs could be restored by a two-hit approach that blocks the EGFR pathway in multiple locations. In accordance with previous reports (Di Nicolantonio *et al*, 2008; Souglakos *et al*, 2009), we found that *BRAF* mutation predicted resistance to cetuximab treatment in the subpopulation of patients that have received this kind of therapy. Furthermore, the prognostic impact of *BRAF* mutation remains significant not only in the whole of the study but also in the subpopulation of patients that have not received an anti-EGFR moAbs.

In summary, *BRA*F V600E mutations, which are correlated with MSI-H status and cyclin D1 overexpression, characterise a subgroup of patients with poor prognosis. The findings reported in this study confirm our previous observations (Souglakos *et al*, 2009) as well as from other groups (Samowitz *et al*, 2005). Nevertheless, they need to be formally confirmed in prospective randomised clinical trials, as this subgroup of patients might justify foregoing approved treatments in favour of investigational ones. Furthermore, the adverse significance of *BRAF* mutations could be used to stratify patients in future clinical trials because these patients carry a significant higher risk of progression and death due to the disease.

## Figures and Tables

**Table 1:** Characteristics of enrolled patients and univariate analysis for PFS and OS

	***N***		**First-line progression-free survival**				**Overall survival**	
**Feature**	**144**	**%**	**Median (months)**	**HR (95% CI)**	***P*-value**	**Median (months)**	**HR (95% CI)**	***P*-value**
Median age (range)	64 (23–81)							
⩽65 years	76	53	9.7	1.3 (0.9–1.8)	0.1	32.2	1.1 (0.8–1.7)	0.5
>65 years	68	47	8.1			29.3		
								
Gender								
Male	82	57	8.9	1.3 (0.9–1.8)	0.2	30.2	1.2 (0.8–1.7)	0.5
Female	62	43	10.3			32.1		
								
Stage at diagnosis								
I–III	82	57	9.4	1.1 (0.7–1.7)	0.9	31.3	1.4 (0.8–2.3)	0.2
IV	62	43	8.1			32.1		
								
Tumour location								
Colon	105	73	9.8	1.3 (0.6–2.6)	0.5	29.8	1.7 (0.7–4.6)	0.3
Rectum	39	27	10.4			31.9		
								
Number of treatment lines	Median: 3							
1						29.8		
2						34.8	0.8 (0.5–1.5)	0.6
3						44.2	0.4 (0.2–0.0)	0.02
								
Histological grade								
I–II	107	74	12.2	2.0 (1.3–3.2)	0.001	39.2	2.7 (1.6–4.4)	<0.001
III	37	26	7.6			23.8		
								
Adjuvant treatment								
Yes	58	40	9.5	1.2 (0.6–2.5)	0.5	31.6	1.1 (0.6–1.8)	0.9
No	86	60	10.2			31.4		
								
Metastasectomy								
Yes	21	15	24.2	0.5 (0.2–0.7)	<0.001	48.7	0.6 (0.2–0.9)	0.03
No	123	85	9.1			31.4		
								
*BRAF* status								
Mutant	12	8	2.7	2.9 (1.6–5.4)	<0.001	14.0	4.3 (2.3–8.2)	<0.001
Wt	132	92	9.8			30.3		
								
MSI status								
High	22	15	9.7	1.0 (0.7–1.7)	0.8	21.3	1.3 (0.8–2.1)	0.3
Stable	122	85	10.4			30.2		
								
Cyclin D1								
Overexpression	26	18	8.3	0.8 (0.5–1.4)	0.1	21.7	1.1 (0.6–1.8)	0.7
Weak expression	63	44	9.1			29.1		
No expression	55	38	11.5			30.3		

Abbreviation: MSI=microsatellite instability.

**Table 2:** *BRAF* mutation and MSI status and correlations with PFS and OS

	**MSI-H, *n*=22 (15%)**		**MSS, *n*=122 (85%)**		
**Total (*n*=144)**	***N* (%)**	**Median (months)**	***N* (%)**	**Median (months)**	***P*-value**
*Progression-free survival*					
*BRAF* mutant, *n*=12 (8%)	10 (45)	3.1	2 (1.6)	1.6	0.003^&^
*BRAF* wt, *n*=132 (92%)	12 (55)	11.4	120 (98.4)	9.7	0.2^@^
*P*-value		0.008^*^		<0.001^#^	
					
*Median overall survival*					
*BRAF* mutant, *n*=12 (8%)	10 (45)	14.5	2 (1.6)	2.6	0.05^&^
*BRAF* wt, *n*=132 (92%)	12 (55)	35.5	120 (98.4)	30.2	0.4^@^
*P*-value		0.004^*^		<0.001^#^	

Abbreviation: MSI=microsatellite instability.

^*^P-value: MSI-H *BRAF* mutant *vs* MSI-H *BRAF* wt.

^#^*P*-value: MSS *BRAF* mutant *vs* MSS *BRAF* wt.

^&^*P-*value: MSI-H *BRAF* mutant *vs* MSS *BRAF* mutant.

^@^*P*-value: MSI-H *BRAF* wt *vs* MSS *BRAF* wt.

**Table 3:** Treatment regimens used in this retrospective study

**First-line regimens**	***N* (out of 144)**	**%**
FOLFOX+Bevacizumab	33	23
FOLFOX+Cetuximab	9	6
FOLFOXIRI	67	46
FOLFIRI	27	19
FOLFOX	8	6
Oxaliplatin-based treatment (first line)	117	82
Irinotecan-based treatment (first line)	94	65
Bevacizumab+chemotherapy (first line)	33	23
Oxaliplatin-based treatment (any line)	128	89
Irinotecan-based treatment (any line)	123	85
Bevacizumab+chemotherapy (any line)	74	51
Cetuximab+chemotherapy (any line)	69	48
Patients treated with all 3 chemotherapy drugs	126	87
Patients treated with all 5 active agents	65	45

Abbreviations: FOLFOX=folinic acid, 5FU, oxaliplatin; FOLFIRI=folinic acid, 5FU, irinotecan; FOLFOXIRI=folinic acid, 5FU, oxaliplatin, irinotecan.

**Table 4:** Correlations of *BRAF* mutations with MSI status and cyclin D1 expression in 144 patients

	**MSI status, *N* (%)**					**Cyclin D1, *N* (%)**		
		**High**	**Stable**		**Overexpressed**	**Weak expression**	**No expression**	
	**No of patients**	**22 (15)**	**122 (85)**	***P*-value** a	**26 (18)**	**63 (44)**	**55 (38)**	***P*-value** a
*BRAF V600E mutation*								
Mutant	12	10 (83)	2 (17)	<0.001	7 (58)	3 (25)	2 (17)	0.001^b^
Wild type	132	12 (9)	120 (91)		19 (14)	60 (46)	53 (40)	

Abbreviation: MSI=microsatellite instability.

a Fisher's exact test.

b Overexpression *vs* weak or no expression.

**Table 5:** Results of multivariate analysis for PFW and OS of 144 patients

	**Hazard ratio**	**95% CI**	***P*-value**
*Progression-free survival*			
*BRAF* (mutant *vs* wt)	2.8	(1.4–5.7)	0.004
Tumor grade (3 *vs* 1–2)	2.0	(1.3–3.2)	0.001
Metastasectomy (yes *vs* no)	0.5	(0.3–0.8)	0.004
			
*Overall survival*			
*BRAF* (mutant *vs* wt)	5.3	(2.5–11.3)	<0.001
Tumor grade (3 *vs* 1–2)	2.6	(1.6–4.4)	<0.001
Metastasectomy (yes *vs* no)	0.6	(0.3–0.9)	0.02
Number of treatment lines (first *vs* ⩾second line)	0.4	(0.2–0.7)	0.009

Abbreviation: CI=Confidence interval.
